# Surveillance for substandard and falsified medicines by local faith-based organizations in 13 low- and middle-income countries using the GPHF Minilab

**DOI:** 10.1038/s41598-022-17123-0

**Published:** 2022-07-30

**Authors:** Gesa Gnegel, Christine Häfele-Abah, Richard Neci, Difäm-EPN Minilab Network, Markous Alladjaba, Micha Lächele, Neenodji Grace, Ndilta Djekadoum, Julien Basile Gounouman, Servilien Mpawenimana, Egide Muziganyi, Anastasie Mukamanzi, Jean Claude Zawadi, Tambo Ajong Cletus, Ndze Edward Ngah, Bishnu Chakraborty, Georges Munguakonkwa Mutombo, Sr Jane Frances Chioke, Esther Okpan, Juliet Ngene, Emmanuel Higenyi, Priscilla Agiro, Titus Uggi, Tumaini Petro Anderson, Pamella Ndakengurutse, Emmanuel Ndayikeza, Stephen Kigera, Mildred Wanyama, Frederick Sowah, Fredrick Kachiponde, Folita Malanda, Dina Pecke Julienne, Fidelis Nyaah, Manyi Pattinora Dohnji, Richard Neci, Gesa Gnegel, Christine Häfele-Abah, Lutz Heide

**Affiliations:** 1 Pharmaceutical Institute, Eberhard Karls University Tuebingen, Tuebingen, Germany; 2 German Institute for Medical Mission (Difäm), Tübingen, Germany; 3 Ecumenical Pharmaceutical Network (EPN), Nairobi, Kenya

**Keywords:** Public health, Drug regulation

## Abstract

This study evaluates the use of the Global Pharma Health Fund (GPHF) Minilab for medicine quality screening by 16 faith-based drug supply organizations located in 13 low- and middle-income countries. The study period included the year before the COVID-19 pandemic (2019) and the first year of the pandemic (2020). In total 1,919 medicine samples were screened using the GPHF Minilab, and samples showing serious quality deficiencies were subjected to compendial analysis in fully equipped laboratories. Thirty-four (1.8%) of the samples were found not to contain the declared active pharmaceutical ingredient (API), or less than 50% of the declared API, or undeclared APIs, and probably represented falsified products. Fifty-four (2.8%) of the samples were reported as substandard, although the true number of substandard medicines may have been higher due to the limited sensitivity of the GPHF Minilab. The number of probably falsified products increased during the COVID-19 pandemic, especially due to falsified preparations of chloroquine; chloroquine had been incorrectly advocated as treatment for COVID-19. The reports from this project resulted in four international WHO Medical Product Alerts and several national alerts. Within this project, the costs for GPHF Minilab analysis resulted as 25.85 € per sample. Medicine quality screening with the GPHF Minilab is a cost-effective way to contribute to the global surveillance for substandard and falsified medical products.

## Introduction

Substandard and falsified (SF) medicines pose a severe risk for patients worldwide, especially in low- and middle-income countries (LMICs) where 10.5% of all medicines have been estimated by the World Health Organization (WHO) to be substandard or falsified^1,2^. According to the current definitions of the WHO, falsified medicines are products which “deliberately/fraudulently misrepresent their identity, composition, or source”^1^. Substandard medicines are products which, without deliberate/fraudulent intent, fail to meet their quality standards^1^, e.g. due to poor manufacturing practice, poor packaging, or inappropriate transportation and storage conditions. SF medicines frequently fail to cure the patients and may thereby cause prolonged illness or even death. They may also lead to severe adverse effects^1–5^. In addition, under-dosed anti-infectives contribute to the global emergence of antimicrobial resistance^1,2,6^.

The SARS-CoV-2 pandemic has affected the supply of medicines especially in LMICs, both by an increased demand for medical products for the treatment and prevention of COVID-19 and by the disruption of supply chains worldwide^7–11^. This has created prospects for criminals to introduce illegal products into the supply chains. An imminent increase in the occurrence of SF medicines was predicted early in the course of the pandemic^12^. Indeed, reports on the seizure of huge amounts of SF medical products, including products related to COVID-19, have been published shortly thereafter^13^.

Pharmaceutical analysis for the identification of SF medicines is usually carried out according to the methods of pharmacopoeias. These analytical methods, also called “compendial analysis”, require well-equipped laboratories and highly educated personnel, and are costly and time-consuming^14^. In many low-resource settings, the timely performance of compendial analyses is challenging or even impossible. Therefore, the introduction of simple, low-cost screening technologies which allow the rapid detection of SF medicines, and their subsequent removal from supply chains, represent one suitable intervention in this context^15–20^. However, a recent publication comparing available screening technologies concluded that “the evaluation of medicine quality screening devices in laboratory and in real-life-settings is [still] in its infancy”^19^, and stated that more research is required to explore the respective benefits, prerequisites and limitations of such instruments^20^.

With almost 900 devices distributed to 98 countries, the Global Pharma Health Fund (GPHF) Minilab is the most frequently used screening technology for medicine quality in LMICs^18,21,22^. According to the GPHF Minilab manual^23^, the analysis comprises three steps: a visual inspection of label, packaging and product; a simplified disintegration test; and a thin-layer chromatographic analysis for qualitative and semi-quantitative examination of the active pharmaceutical ingredients (APIs)^23^. While the GPHF Minilab was found to be highly sensitive and specific in the identification of products which do not contain the stated API, it is less sensitive in the detection of products which contain an insufficient amount of the API, or show insufficient dissolution of the API^24,25^.

In 2015 a medicine quality study^26^ was carried out by ten faith-based drug supply organizations located in seven African and Asian countries which were using the GPHF Minilab. Each organization collected medicine samples from private local medicine outlets, including the informal sector, within a six month period. A total of 869 samples were collected and tested. Samples failing the GPHF Minilab analysis were subsequently investigated by compendial analysis in international laboratories. This study resulted in the identification of 12 samples which did not contain the stated API^26^. The successful completion of that study encouraged the German Institute for Medical Mission (Difäm; Tübingen, Germany), and the Ecumenical Pharmaceutical Network (EPN; Nairobi, Kenya) to further expand the use of the GPHF Minilab for basic medicine quality screening by faith-based drug supply organizations in Africa and Asia. The resulting Difäm-EPN Minilab Network comprised 16 member organizations in the period between January 2019 and December 2020. All of them hold valid licenses in their countries, allowing them to procure and distribute medicines to faith-based healthcare facilities. They routinely use the GPHF Minilab for basic quality testing of selected medicines procured for distribution in their organization, and of a certain number of samples from external sources, such as informal vendors, for comparison.

The present report analyses the operation of this Network from January 2019 to December 2020. We purposefully included the year before the COVID-19 pandemic, and the first year of the pandemic, to allow some insight into the effect of the pandemic on the occurrence of SF medicines. In this period, the Network analyzed approximately 2000 medicine samples. We here report on the numbers and types of identified SF medicines, and provide information on costs and organizational requirements for this approach of a low-cost, basic medicine quality screening by local organizations in LMICs. The present study was not designed as a study of the prevalence of SF medicines with a prospective, systematic sampling design, but it reports the results of a routine use of the GPHF Minilab in medicine quality assurance by the involved organizations, and the requirements therefor.

## Methods

### Study design

Starting from 2010 and with support from the faith-based aid organization Bread for the World (Berlin, Germany), Difäm has provided drug supply organizations (DSOs) in Africa and India with GPHF Minilabs and trained them in their use. In 2019 and 2020, the resulting Difäm-EPN Minilab Network comprised 16 member organizations who routinely used the GPHF Minilab for medicine quality screening. These DSOs report all test results to Difäm in form of standardized Excel tables which are filled and submitted every three months. However, the DSOs are encouraged to report samples failing Minilab analysis to Difäm immediately. Difäm organizes confirmatory compendial testing (see below) for samples for which Minilab analysis had indicated serious quality deficiencies. Furthermore, Difäm provides the DSOs with standards, reagents and equipment required for the operation of the Minilabs, and organizes trainings and network meetings. Therefore, the results of the Minilab testing by the network partners, and of the confirmatory compendial analyses, as well as data on the requirements of funds and materials for the network operation are available at Difäm. For the present study, a research pharmacist (G.G.) retrospectively analyzed these data for the period January 2019 and December 2020, including the results of all samples which had been tested by the network members in this period.

### Location of the involved organizations, and qualification of the personnel responsible for GPHF Minilab screening

The 16 DSOs are located in 13 LMICs in Africa and Asia (see “Results” section). All DSOs employed at least one pharmacist or pharmacy technician, and at least two staff members of each DSO had been trained in the use of the GPHF Minilab^26^. Fourteen out of the 16 DSOs were active during the entire investigated period, one organization left the Network in 2019, another one joined in 2020.

### Sample collection

In the Difäm-EPN Minilab Network, each DSO signs a Memorandum of Understanding pledging to collect and analyze at least 75 medicine samples yearly; a smaller number than 75 is considered inefficient in view of the requirements for the regular provision of consumables, equipment and training. However, for one relatively small organization, a number of 50 samples was agreed.

The DSOs are requested to collect medicines for which Minilab protocols exist. The GPHF Minilab manual^23^ contains protocols for the analysis of 100 APIs mainly in the forms of tablets, capsules and injectables, as well as for frequently used fixed combinations of these APIs. Minilab protocols for seven further APIs have been developed in 2021 and 2022, i.e. after the period under investigation^27^.

Difäm and EPN provide guidance to the DSOs for medicine procurement in accordance to WHO recommendations^28^, including supplier selection and prequalification. Respective trainings are offered to the DSOs on a regular basis. Depending on the resources of the respective DSO, and on the possibilities and limitations for medicine procurement in the respective country, the degree of adherence to the WHO recommendation varies between DSOs.

The process of sample collection is left in the responsibility of the individual DSOs. The DSOs use the Minilab primarily to screen the quality of those medicines which they routinely procure and distribute to their member health facilities and patients. The sources of these medicines include faith-based, governmental, and private vendors, i.e. licensed medicine sellers. A very small number of samples were received by the DSOs as donations from foreign charitable organizations.

Some additional samples were collected from health facilities, obtained either during routine supervisory visits by DSO staff or sent by health facilities to the DSOs for testing purposes, e.g. in cases of doubt about the quality of the respective product. In addition, Difäm encouraged the DSOs to collect and analyze some samples from external sources such as informal vendors. Previous studies have shown that informal vendors are an especially important route of entry of SF medicines into the market^24,29^, and analyzing medicine samples from informal vendors is a useful way to sensitize the DSOs towards the occurrence of such SF medicines. A refund of the purchase costs of such samples was offered by Difäm to the DSOs, but the number of samples collected from external sources was left to the DSOs to decide. The DSOs were free to use either an open or a mystery shopper approach when purchasing from informal vendors; the mystery shopper approach was most frequently chosen.

Prior to the Minilab testing, collected medicine samples were stored in the medicine storage facilities of the respective DSOs under appropriate conditions.

### Minilab analysis

According to the GPHF Minilab manual^23^, medicine samples were investigated by visual inspection, disintegration testing (if applicable) and thin-layer chromatography (TLC). In most organizations, all three steps were performed by the same person, hence no blinding was applied.

Visual inspection was carried out as specified in the chapter “Visual Inspection” of the GPHF Minilab manual^23^. Especially, product labelling was checked for completeness and plausibility of information, and correct spelling. The primary packaging was examined for adequate protection of the medicine, and the dosage units were screened for visual deficiencies.

Disintegration testing was carried out as specified in the respective chapter of the GPHF Minilab manual^23^. It should be noted that this is not a compendial test but a simplified procedure, and that no thermostated device is used. Six tablets and capsules were immersed in a flask containing 100 mL water of 37 °C, and the liquid was stirred or shaken from time to time. Immediate release tablets and capsules were considered as compliant if all six units fully disintegrated within 30 min, while slow-release and enteric-coated products have to withstand this test and must not disintegrate before 30 min. This test is not applicable for injectables, dry syrups and chewable tablets.

TLC analysis: Sample and reference tablets were crushed (capsules were opened, injectables and dry syrups were used as such) and extracted with a defined volume of the solvent described in the respective monograph of the GPHF Minilab manual. After a dilution step, 2 µL of the solution were applied to a TLC plate (Merck silica gel 60 F254, 0.2 mm thickness, 5 × 10 cm) using a microcapillary. On each plate, two reference solutions were applied, one corresponding to 100% of the stated amount of the respective API, the other corresponding to 80%. In addition, two spots of sample solution were applied on the plate. After development of the plate in the mobile phase described in the respective monograph of the manual, the spots were detected using UV light (254 or 366 nm) or a chemical staining method (iodine vapor; ninhydrin or sulfuric acid solutions) as described in the GPHF Minilab manual. The sample was rated compliant if the sample spots showed the same travel distance (relative retention factor) as well as the same color, size, and shape as the reference spots, were not weaker than the 80% reference spot and did not show additional spots indicating the presence of undeclared compounds or contaminants.

### Additional/confirmatory testing

When Minilab testing revealed severe quality deficiencies (such as absence of the declared API; presence of a non-declared API; grossly insufficient amount of the declared API; or disintegration times of > 2 h), the DSOs reported these to the investigators in Tübingen using a standardized Microsoft Word form, together with photographs of the product and the developed TLC plate. The reports were checked by G.G. and C.H. to exclude possible false alerts, e.g., due to misinterpretation of TLC results, and further investigations were initiated. In eight cases G.G. and C.H. considered that the Minilab analysis by the first partner organization did not allow an unequivocal classification as compliant or non-compliant. In these cases, the suspect samples were sent to a second partner organization for re-testing. If test or re-test clearly revealed severe quality deficiencies, samples were forwarded to a fully equipped laboratory for compendial analysis using commercial courier services. In the study period, compendial analysis was conducted in 25 cases, either at the WHO prequalified laboratory of Mission for Essential Drugs and Supplies (MEDS; Nairobi, Kenya) or in the Pharmaceutical Institute of Tübingen University. No blinding was employed, i.e., persons performing the re-tests or compendial analyses were aware of previous testing results. Compendial testing was carried out following the current monographs of the United States Pharmacopeia (USP) or British Pharmacopoeia (BP), or according to in-house procedures established by MEDS if no respective USP or BP monograph was available. Identity and assay tests were performed in all 25 compendial analyses. Dissolution testing was carried out for solid oral dosage forms; however, it was omitted for samples not containing the declared active ingredient, and for samples containing less than 80% of the stated amount of API. Further tests, e.g., for related products, were performed as necessary. In cases where the identification of non-declared compounds was required, high resolution mass spectroscopy was performed^30^.

Due to budget constraints, samples which showed minor quality deficiencies in the Minilab testing (such as spelling mistakes on the label or a disintegration time between 30 min and 2 h) were not submitted to compendial analysis. In some cases, the re-test and/or compendial analysis could not be performed due to an insufficient amount of remaining dosage units.

### Routine documentation of samples, of test results and of costs

The results of all performed analyses were sent by the DSOs to the research pharmacists at Tübingen quarterly. In a standardized drop-down Microsoft Excel sheet, the source of the collected sample, product name, API, strength, manufacturing date, expiration date, batch number, name of stated manufacturer, country of manufacturing, and the month of testing were reported. Receipts and invoices of all expenditures related to the activities of the Difäm-EPN Minilab Network were collected, and costs were analyzed by the research pharmacist (G.G.).

### Definitions

“Substandard” and “falsified” medical products were defined as suggested by WHO^1^. Since many but not all detected deficient products could be forwarded to an unequivocal compendial analysis, and in many cases the (stated) manufacturers could not be reached to confirm suspected falsifications, we use the cautious terms “probably falsified” and “probably substandard” for most of the results of the present evaluation. As suggested by Hauk et al.^31^, samples containing no API, an incorrect API, or less than 50% of the stated API (without presence of degradation products) were rated as “probably falsified”. The twelve African countries investigated in this research were assigned to five geographic regions of Africa as defined by the Organisation of African Unity (the predecessor of the African Union) in 1976 (CM/Res.464QCXVI)^32^. For the purpose of this research, “sample” is defined as a medical product of a specific brand and batch, collected at the same time and same place and subsequently subjected to one or several analyses.

### Statistical methods

Data collection and basic evaluation were performed using Excel by Microsoft Office Professional Plus 2016. Statistical analyses were conducted using MedCalc (MedCalc Software, Ostend, Belgium)^33^. Comparisons of proportions were evaluated by the two-sided N-1 Chi-squared test as recommended by Campbell and Richardson for two-by-two tables with small sample sizes^34,35^. All p-values < 0.05 were considered significant.

## Results

### Overview of collected samples

**Figure 1:**
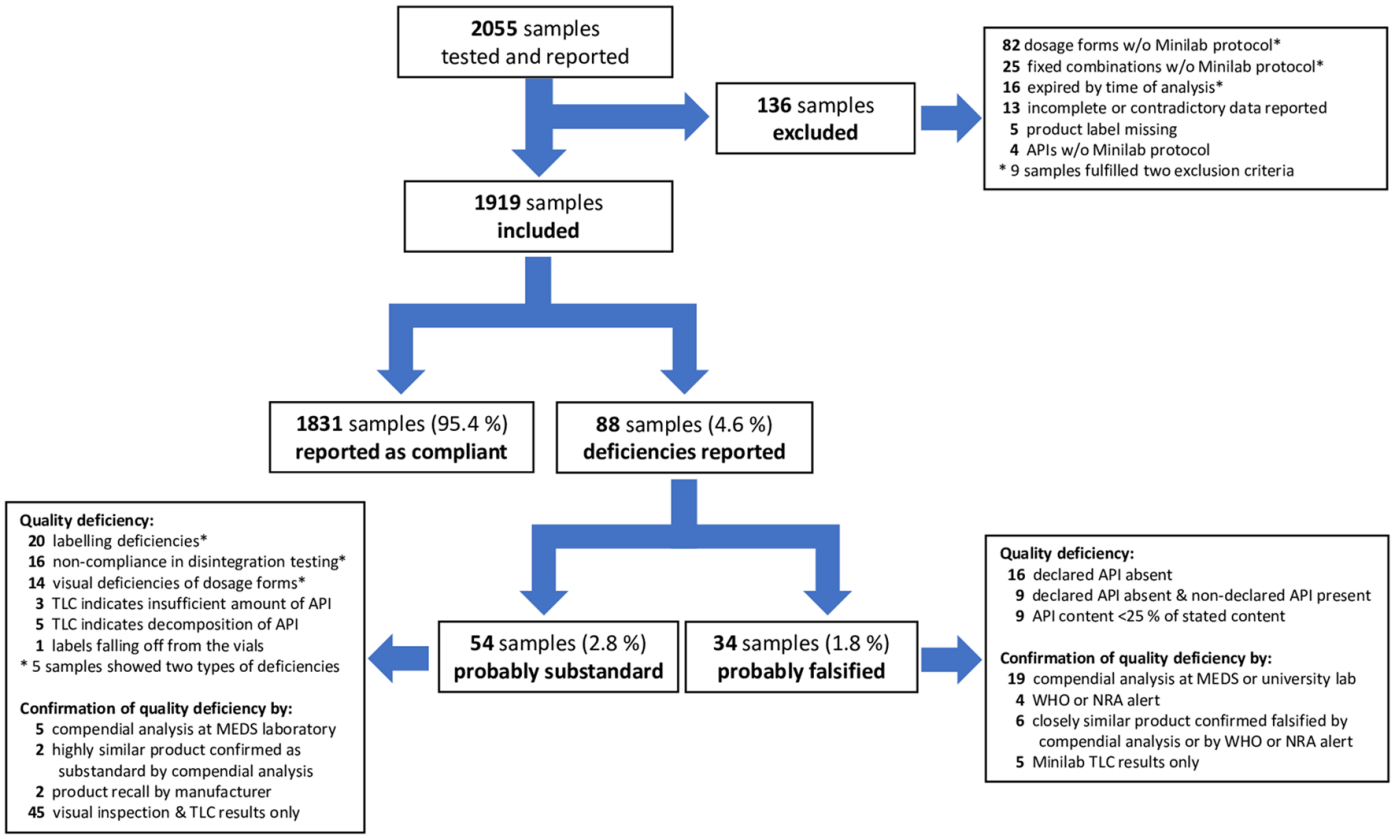
Flow chart showing the evaluation of the reported medicine quality data.

As shown in Fig. 1, 2055 samples were tested and reported in the course of this study. Each of the eight samples sent to a second partner organization for re-testing (see “Methods”) was counted as a single sample. A total of 136 samples were excluded from the present data analysis, most frequently because they represented oral liquid dosage forms. With the exception of protocols for dry syrups containing artemether/lumefantrine, amoxicillin or amoxicillin/clavulanic acid, no Minilab protocols for oral liquid dosage forms exist, as the excipients present in syrups and suspension may interfere with the TLC analysis and may preclude the reliable interpretation of the result. As shown in Fig. 1, analytical results correctly based on GPHF Minilab protocols were reported for 1,919 samples, and these were included into the data analysis.

Samples were collected and analyzed in 13 countries by 16 faith-based DSOs, as summarized in Table 1. Fifteen of these organizations are located in sub-Saharan Africa, one in India. Of the 1919 samples included in the data analysis, 1591 (82.9%) were collected from the own stock of these DSOs, or from private vendors. Private vendors are commercial sources from which these organizations purchase medicines for distribution to health facilities, therefore the two categories “own stock” and “private vendors” are combined in Table 1. A total of 205 samples (10.7%) were collected from health facilities. Only 10 samples (0.5%) were products donated to the DSOs. Further 111 samples (5.8%) were collected from informal vendors, with 106 of these collected in four of the five included countries of Central Africa.

**Table 1:** Overview of collected medicine samples, and results of analysis.

Region and country of collection	Sources of samples				Total no. of samples included	Results of analysis			
	Private vendors or own stock	Health facilities	Donations or unknown	Informal vendors		No. of probably falsified samples	%	No. of probably substandard samples	%
**East Africa**	**152**	**5**	**1**	**3**	**161**	**0**	**0.0%**	**3**	**1.9%**
Kenya	43	0	0	0	43	0	0.0%	3	7.0%
Rwanda	9	0	0	0	9	0	0.0%	0	0.0%
Tanzania	14	5	1	0	20	0	0.0%	0	0.0%
Uganda	86	0	0	3	89	0	0.0%	0	0.0%
**Central Africa**	**1126**	**68**	**0**	**106**	**1300**	**31**	**2.4%**	**48**	**3.7%**
Burundi	241	1	0	0	242	0	0.0%	7	2.9%
Cameroon	686	19	0	40	745	11	1.5%	20	2.7
Central African Rep	22	11	0	17	50	3	6.0%	8	16.0%
DR Congo	142	11	0	10	163	6	3.7%	3	1.8%
Chad	35	26	0	39	100	11	11.0%	10	10.0%
**West Africa**	**171**	**103**	**0**	**2**	**276**	**3**	**1.1%**	**3**	**1.1%**
Ghana	0	27	0	0	27	0	0.0%	0	0.0%
Nigeria	171	76	0	2	249	3	1.2%	3	1.2%
**Southern Africa**	**78**	**29**	**11**	**0**	**118**	**0**	**0.0%**	**0**	**0.0%**
Malawi*	78	29	11	0	118	0	0.0%	0	0.0%
**Southeast Asia**	**64**	**0**	**0**	**0**	**64**	**0**	**0.0%**	**0**	**0.0%**
India	64	0	0	0	64	0	0.0%	0	0.0%
**Total**	**1,591**	**205**	**12**	**111**	**1919**	**34**	**1.77%**	**54**	**2.81%**

Three faith-based drug supply organizations in Cameroon contributed to this study, and two in the Democratic Republic of the Congo (DRC).

*For two samples from Malawi, the source is unknown.

The different organizations involved in this study had different staffing capacities for their contribution to the Minilab surveillance project. The most active organization, located in Cameroon, contributed 512 samples. The partner organization in Rwanda joined the surveillance only in the second project year, and therefore contributed only nine samples.

**Figure 2:**
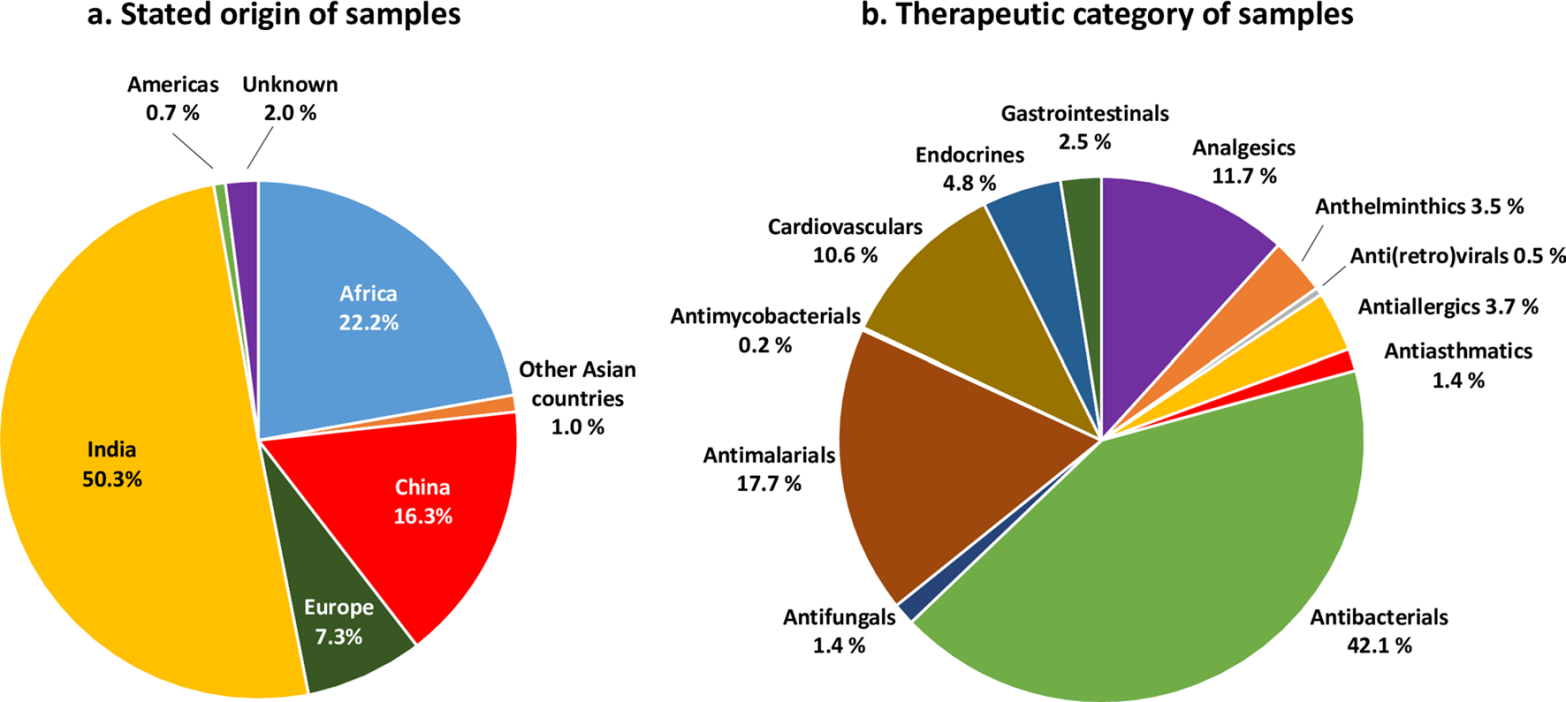
Stated origin (**a**) and therapeutic categories (**b**) of the 1919 included samples.

The stated countries of origin of the included medicine samples are depicted in Fig. 2a. Half of the samples (966 samples; 50.3%) were stated to be produced in India, and 313 (16.3%) in China. Further 426 samples (22.2%) were stated to be produced in Africa, with Nigeria (159 samples; 8.3%), Kenya (98 samples; 5.1%) and Uganda (72 samples; 3.8%) as the most important African producer countries.

As shown in Fig. 2b, 1252 samples (65.2%) were medicines for the treatment of infectious diseases, with antibacterials (808 samples; 42.1%) and antimalarials (339 samples; 17.7%) as most frequent categories. Among the medicines for non-communicable diseases, analgesics were included most frequently (225 samples; 11.7%). The most frequently tested dosage forms were tablets (1467 samples; 76.4%), followed by injections (250 samples; 13.0%), capsules (168 samples; 8.8%), and dry syrups (34 samples; 1.8%).

In total, 71 different APIs were tested according to the protocols of the GPHF Minilab manual. A detailed overview of the different APIs and dosage forms of the medicines included into the data analysis is given in Supplementary Table S1.

### Results of sample analysis

A research pharmacist (G.G.) cross-checked the data reported by the partner organizations from Africa and Asia, and the categorization of the results as compliant or non-compliant by the partners. Corrections by the research pharmacist were required only in 15 cases (0.8%). Most frequently (six cases), a failure in disintegration testing had been incorrectly reported for modified release tablets; as stated in the GPHF Minilab manual^23^, these are in fact not expected to disintegrate under the test conditions specified in the Minilab protocol.

Samples which showed major quality deficiencies in the TLC analysis (absence of stated API, presence of undeclared substances, underdosage of declared API) and/or in the disintegration test (i.e., disintegration time > 2 h) were sent for compendial analysis to MEDS or Tübingen University. In eight ambiguous cases (0.4%) a re-test by a second Network member was sought. Three of these cases were found to be compliant in the Minilab re-test, in the other five cases further investigation was considered necessary. Subsequently conducted compendial analysis at the MEDS laboratory revealed that two suspected products were compliant with the specifications while three were not.

**Figure 3:**
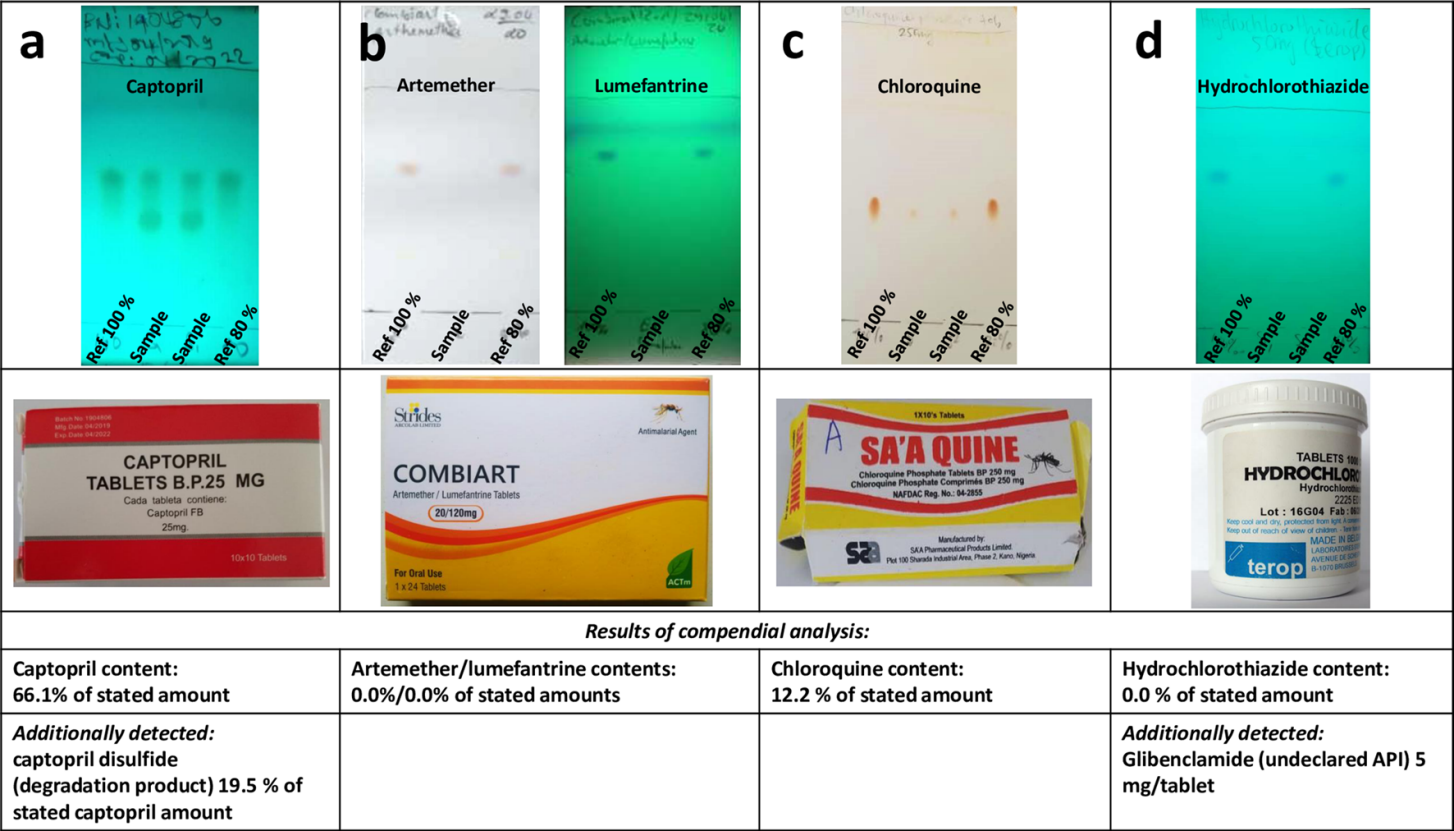
Examples of TLC analysis of samples of the present study, showing (**a**) decomposition of the API; (**b**) absence of the declared APIs; (**c**) API content 12.2% of the stated amount; (**d**) absence of the declared API, and presence of a non-declared API (the non-declared API glibenclamide is not visible in the depicted TLC plate, but was discovered by the local partner in an additional, specific TLC analysis for glibenclamide, prompted by the observed hypoglycemic effect of the falsified medication^36^). (Photos: Gesa Gnegel, Lutz Heide and Difäm-EPN Minilab Network).

After these corrections, eventually 1831 samples (95.4%) were reported as compliant, while for 88 samples (4.6%) quality deficiencies were reported and these were considered as SF products (Fig. 1). Of the 88 samples reported to show deficiencies, 34 (1.8% of evaluated samples) were rated as probably falsified by the research pharmacist: for 16 of these 34 samples, TLC analysis showed that the stated API was absent. In another 9 samples the stated API was absent, but they contained a different, undeclared API. In the remaining 9 cases, visual analysis of the TLC results suggested that the API was present in a much smaller amount than stated on the label, and indeed compendial analysis confirmed for these samples that the API content was < 25% of the stated amount (see Table 2), and at the same time no decomposition products were detected. Examples of TLC results for different types of quality deficiencies are depicted in Fig. 3.

**Table 2:** Medicine samples identified in this study as probably falsified.

No.	Country of discovery	Declared active APIs, and dosage form	Stated country of origin	API content (% of stated amount); + undeclared APIs
1	Cameroon	Ampicillin/cloxacillin sodium capsules	India	0%/0%
2	Chad	Artemether/lumefantrine tablets	India	0%/0%
3	DR Congo	Ceftriaxone sodium inj. 1 g	Spain	23.5%
4	DR Congo	Ceftriaxone sodium inj. 1 g	Spain	23.8%
5	DR Congo	Ceftriaxone sodium inj. 1 g	Spain	< 23% (weight of vial content = 230 mg)
6	DR Congo	Chloroquine tablets	Kenya	0%; + 126.5 mg metronidazole
7	Cameroon	Chloroquine phosphate tablets	India	0%
8	Nigeria	Chloroquine phosphate tablets	Nigeria	0%
9	Cameroon	Chloroquine phosphate tablets	Nigeria	0%
10	Cameroon	Chloroquine phosphate tablets	China	21.7%
11	Cameroon	Chloroquine phosphate tablets	China	0%; + 14.1 mg/tablet metronidazole
12 & 13	Cameroon	Chloroquine phosphate tablets	China	0%; + 35.7 mg/tablet paracetamol
14	Cameroon	Chloroquine phosphate tablets	China	0%; + 14.6 mg/tablet metronidazole + 1.6 mg/tablet paracetamol
15	Cameroon	Chloroquine phosphate tablets	Nigeria	12.2%
16	Cameroon	Hydrochlorothiazide tablets	Belgium	0%; + 5 mg/tablet glibenclamide
17	Cameroon	Paracetamol/diclofenac sodium tablets	India	95.2% paracetamol/0% diclofenac
18	Nigeria	Proguanil tablets	Malta	< 25% (estimate from TLC)
19	DR Congo	Quinine tablets	India	0%
20	Chad	Quinine tablets	Nigeria	0%
21	Central Afr. Rep	Quinine sulphate tablets	Nigeria	0%
22	Central Afr. Rep	Quinine sulphate tablets	Nigeria	0%
23	Central Afr. Rep	Quinine sulphate tablets	Bulgaria	0%
24	Chad	Quinine sulphate tablets	Cyprus	0%; + 12 mg/tablet chloroquine
25	DR Congo	Quinine sulphate tablets	Uganda	0%
26	Chad	Quinine sulphate tablets	Norway	0%
27	Chad	Sulfamethoxazole/trimethoprim tablets	Nigeria	0%/0%
28	Nigeria	Sulfamethoxazole/trimethoprim tablets	Nigeria	0%/0%
29	Chad	Sulfamethoxazole/trimethoprim tablets	Not stated	0%/0% + paracetamol (TLC analysis)
30	Chad	Sulfamethoxazole/trimethoprim tablets	Nigeria	0%/0%
31	Chad	Sulfamethoxazole/trimethoprim tablets	Nigeria	0%/0%
32	Chad	Sulfamethoxazole/trimethoprim tablets	Nigeria	47.7%/21.2%
33	Chad	Sulfamethoxazole/trimethoprim tablets	Nigeria	17.6%/16.3%
34	Chad	Sulfamethoxazole/trimethoprim tablets	Nigeria	< 25%/< 25% (estimate from TLC)

In the cases 12 and 13, two samples of this medicine were identified independently in the course of this study. Supplementary Table S2 provides further details on these samples, including the brand names of the products, batch numbers, expiry dates and names of the stated manufacturers.

For 19 of the 34 samples rated as probably falsified, the quality deficiencies detected in the GPHF Minilab analysis were confirmed by compendial analysis in the WHO-prequalified laboratory of MEDS or in the laboratory of Tübingen University. For another four samples, compendial analysis was considered unnecessary since reports published by WHO or by a national medicines regulatory authority confirmed that these samples were falsified. For six samples, closely related products had previously been identified as falsified by compendial analysis or an (inter-)national alert. For the remaining five samples, the low number of tablets remaining after the Minilab testing did not allow a confirmation by compendial analysis, but the evidence from TLC analysis and packaging analysis was considered unequivocal.

In total 54 samples (2.8% of evaluated samples) were rated probably substandard (Fig. 2) because of one or several of the following reasons. The most common reason (20 samples) was deficient labelling, such as missing batch numbers or orthographic mistakes, however without conclusive evidence for falsification as described by Hauk et al.^31^. Fourteen samples showed visual deficiencies of the dosage forms, such as discolorations or cracks in case of tablets, or agglomeration of capsules. Sixteen samples showed non-compliance in disintegration testing. In two of these cases, the tablets had not disintegrated even after two days. The faith-based drug supply organization decided to contact the local manufacturer, presented the test result, and the manufacturer thereupon issued a product recall, as depicted in Supplementary Fig. S1.

In three out of the 54 samples rated as probably substandard, TLC analysis indicated an insufficient amount of the API, estimated to be in the range of 50–80% of the declared amount by visual inspection of the TLC plate. Unfortunately, in these three cases the sample size was insufficient to allow for compendial analysis. In five further samples, representing different batches of captopril tablets from two manufacturers, TLC analysis indicated decomposition of the API (Fig. 3a). Compendial analysis conducted for one sample from each manufacturer respectively proved API contents of only 66.1% and 50.7% of the declared amount, as well as elevated, non-compliant quantities of the decomposition product captopril disulfide.

The limited funds available for the present project did not permit to subject all samples rated as probably substandard to compendial analysis.

### Samples identified as probably falsified in this study

Table 2 lists the 34 samples rated as probably falsified, with their declared APIs, their countries of discovery, their stated country of manufacture, and the result of their chemical analysis. Supplementary Table S2 provides further details on these samples, including the brand names of the products, batch numbers, expiry dates and names of the stated manufacturers.

Ten of these 34 samples were labeled to contain chloroquine as API, eight to contain quinine, and another eight to contain sulfamethoxazole/trimethoprim. Out of the 34 probably falsified medicines 32 were anti-infectives. Probably falsified samples were found only in five of the 13 countries where this study was conducted, i.e., in Cameroon, Chad, DRC, and CAR (Central Africa) and in Nigeria (West Africa). Out of the 111 medicines collected from informal vendors, 14 (12.6%) were rated as probably falsified, contrasting to 20 (i.e., only 1.1%) out of the 1,808 medicines from legal sources (p < 0.0001). Out of the 970 medicines collected from the own stock of the participating faith-based DSOs, only three (0.3%) were rated as probably falsified, indicating a largely successful product and supplier selection by the DSOs.

Fifteen of the 34 probably falsified samples were stated to be produced in Africa (13 of these in Nigeria), and the others in Europe (8), India (5), and China (5). For one sample no country of manufacture was indicated. However, the manufacturer and the country of origin stated on the label of a falsified medicine may obviously be incorrect. Some manufacturers named in Supplementary Table S2, such as Strides Arcolab, India, have an excellent international reputation which falsifiers may have criminally misused in the labelling of their falsified medicines. For the artemether/lumefantrine preparation listed in Supplementary Table S2, Strides Arcolab confirmed to the authors and to WHO that this product is a falsification. Some other manufacturers listed in Supplementary Table S2, such as “Enitop Pharmaceuticals Nig. Ltd” and “Pharmachim Bulgaria” are non-existing companies^37^.

Notably, out of the 34 probably falsified medicines, 22 samples (64.7%) were reported to show deficiencies already in visual inspection, such as missing data or mistakes in the labelling, or visible deficiencies of the dosage forms. In contrast, out of the 1885 medicines considered non-falsified, 34 (i.e., only 1.8%) were reported to show visual deficiencies. This difference is statistically significant (p < 0.0001) and emphasizes that careful visual inspection is an important and powerful tool in the screening for falsified medicines^26,38,39^.

### Changes of the occurrence of substandard and falsified medicines in the course of the COVID-19 pandemic

**Figure 4:**
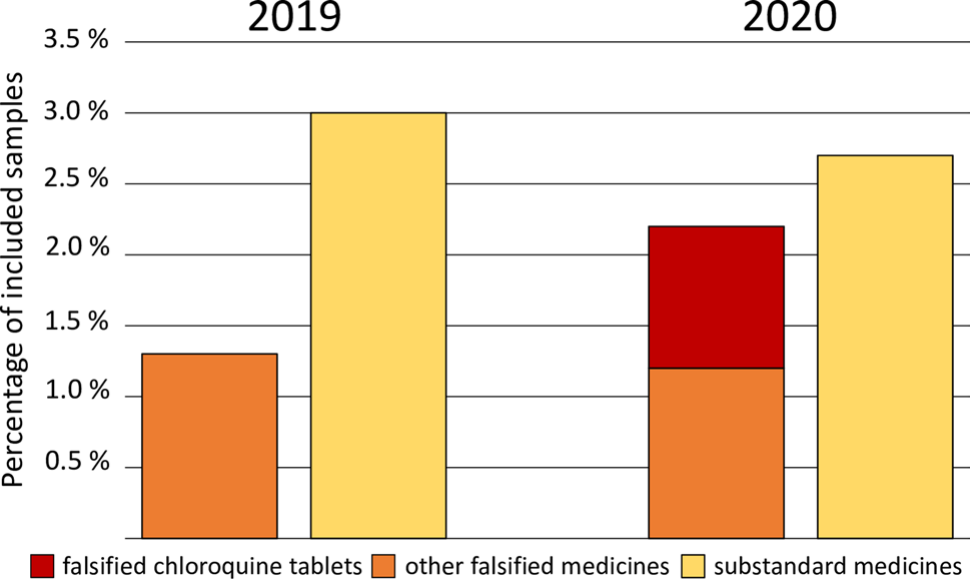
Changes of the occurrence of probably falsified and probably substandard medicines in the course of the COVID-19 pandemic.

Figure 4 compares the number of probably falsified and probably substandard samples reported in the two investigated years. In 2019 (before the pandemic), 11 samples (1.3%) out of 871 were probably falsified. In 2020 (during the pandemic) this increased to 23 (2.2%) out of 1048 samples. Though this difference does not reach statistical significance (p = 0.14), it indicates a trend towards an increase in the occurrence of falsified medicines, as has been predicted at the outset of the pandemic^12^. Notably, the observed increase was nearly entirely due to the occurrence of ten falsified chloroquine samples (nine samples in the first half of 2020, one in the second half).

### Sharing data with stakeholders

All probably falsified samples listed in Table 2 and Supplementary Table 2, as well as four products rated as severely substandard (i.e. amount of the API estimated as 50–80% of the declared amount by visual inspection of the TLC plate; or TLC indicating major decomposition of the API, as shown in Fig. 3a) were reported to the WHO Global Surveillance and Monitoring System for SF Medical Products (Rapid Alert System). These reports were made by G.G., without mentioning the names of the partner organizations to protect their anonymity. WHO decided about further actions, such as contacting the stated manufacturers, informing responsible national regulatory authorities, and in very serious cases publishing international WHO Medical Product Alerts. The medicine quality analyses conducted within this study resulted in the release of four WHO Medical Product Alerts^36,37,40,41^, warning about the cases listed as No. 6, 7, 10–14, 16, 20, 21, 23 and 24 in Table 2/Supplementary Table S2. Several national drug regulatory authorities also published alerts about these cases, e.g. the National Authority for Food and Drug Administration of Nigeria (NAFDAC)^42–44^, the Ministère de la Santé et de la Population of CAR (Supplementary Fig. S2), the Laboratoire National de Contrôle de Qualité des Médicaments et d’Expertise (LANACOME) of Cameroun (Supplementary Fig. S3) and the Drug Regulatory Authority of Pakistan^45^. NAFDAC also published an alert about the case listed as No. 33 in Table 2/Supplementary Table S2^46^.

### Funding requirements for the surveillance for substandard and falsified medicines

As reported by Petersen et al.^26^, the initial provision of GPHF Minilabs for most partners of the Difäm-EPN Minilab Network in the years 2010–2015 had required approximately 5600 US$ per Minilab, and the initial training of the personnel required approximately 2300 US$ per partner organization. As summarized in Table 3, in the present reporting period (2019–2020) external funding was required for consumables, confirmatory compendial analyses, training, and for the research pharmacist (G.G.) at Difäm who also acted as network coordinator (total 0.2 full-time equivalents). All personnel costs required for the local GPHF Minilab analyses were borne by the faith-based DSOs in Africa and Asia themselves. Most of the tested medicine samples were obtained from the own stocks of the participating DSOs, or from the private vendors they used as source of their supplies (Table 1); no external funds were provided for the acquisition of these samples. A refund of the purchase costs for samples from external sources had been offered out of the project budget, but none of the DSOs claimed such refunds in the reporting period, probably due to the comparatively small sums involved. In the original project budget, one yearly network meeting had been foreseen and budgeted at 6,400 € each. Due to the outbreak of the COVID-19 pandemic, however, these meetings were held online, and this budget line remained untouched.

**Table 3:** Funding requirements for the surveillance for substandard and falsified medicines in the reporting period (Jan. 2019–Dec. 2020).

**External funding:**	
14,300 €	Consumables for GPHF Minilab analysis: 41.7% for solvents (procured locally); 32.9% for reference standards and 19.7% for TLC plates, glassware and GPHF Minilab manuals (procured and shipped through TTM Technologie Transfer Marburg e.V.; Coelbe, Germany); 5.6% for packaging and shipment
10,500 €	17 confirmatory compendial analyses at MEDS laboratory, Nairobi (offered at reduced rates)
2,000 €	GPHF Minilab training course in Rwanda
22,800 €	Personnel costs for research pharmacist/network coordinator at Difäm, Tuebingen, Germany (0.2 full-time equivalents)
** = 49,600 €**	**Total external funding** (47,600 € from Bread for the World, Berlin, Germany; 2,000 € from Difäm, Tübingen, Germany)
**Contributions by network participants**	
Staff time for GPHF Minilab analyses and documentation by 16 participating drug supply organizations	
Acquisition costs of medicine samples by 16 participating drug supply organizations	
Purchase of one GPHF Minilab (5,500 €) by one participating drug supply organization, using its own funds	
8 confirmatory compendial analyses at Tuebingen University laboratory, Germany, provided at no charge	

Based on the 1919 samples included into the data analysis (Fig. 1), the costs for consumables for GPHF Minilab analysis (Table 3) resulted as 7.45 € per sample on average. However, this varied between the partners: DSOs testing a small number of samples but including a high number of different APIs required higher costs per sample, e.g. since many reference standards needed to be replaced upon expiry.

Based on the total external funding of 49,600 € (Table 3), the total external costs amounted to 25.85 € per sample. Notably, a compendial analysis of all 1919 samples, even at the reduced rates offered by MEDS for this project (618 € per sample on average), would have costed approximately 1.2 million €, i.e., 23 times more than the actual external funding of the project.

Based on the 34 probably falsified medicines listed in Table 3, the external costs of the identification of one such product resulted as 1459 €.

## Discussion

This study describes procedures, results, costs, and limitations of a routine medicine quality screening using the GPHF Minilab by faith-based DSOs in Africa and Asia. As described recently, the evaluation of medicine quality screening devices, especially in real-life-settings, is still in its infancy^19,20^, and to our knowledge the present study is the first systematic investigation of the routine use of a medicine quality screening device covering a large geographic area (two continents, 13 countries) and a longer period of time (two years).

Since the screening project and its present evaluation were carried out with a minimal budget, many but not all detected deficient products could be forwarded to an unequivocal compendial analysis. Furthermore, it was not possible in many cases to contact the (stated) manufacturers to confirm the observed falsifications. Therefore, we use the cautious terms “probably falsified” and “probably substandard” in the results of the present evaluation. A total of 2,055 medicine samples were screened with the GPHF Minilab by the 16 participating organizations, of which 1,919 samples met the inclusion criteria and were included in the present evaluation (Fig. 1). Of these, 34 samples (1.8%) were classified as probably falsified, since they did not contain the declared API, contained undeclared APIs, or contained less than 50% of the declared API without presence of decomposition products. The quality deficiencies of these samples are summarized in Table 2 and clearly illustrate the huge public health threat posed by such preparations. Similar percentages of falsified medicines as in the present study have been reported in previous studies in LMICs, carrying out compendial analysis for all samples. e.g., Rahman et al.^47^ found 1.1% falsified samples in Bangladesh, and Hauk et al.^31^ found 1.7% in Cameroon and the DR Congo. No falsified medicines were found by Seitzer et al.^48^ among 88 samples from Burkina Faso, Cote d’Ivoire, Ghana and Tanzania. The GPHF Minilab has been proven to be highly sensitive in the detection of the gross deficiencies described above^18,24^, and it appears likely that such deficiencies have been detected reliably and completely in the present GPHF Minilab screening.

In the present study, 54 samples (2.8%) were reported to be probably substandard. Together with the 34 samples rated as probably falsified, this results in 88 SF samples (4.6%). However, we cannot exclude that a considerable number of substandard samples have been missed out, due to the known, limited sensitivity of the GPHF Minilab in the detection of products which contain an insufficient amount of the API, or show insufficient dissolution of the API^24,25^. Notably, an average prevalence of 10.5% SF medicines in LMICs was reported in a review by WHO^1^, and a rate of 18.7% in a review by Ozawa et al.^49^.

In total 38 cases of SF medicines from the present study have been reported to the WHO. Four international Medical Product Alerts were subsequently issued by WHO^36,37,40,41^, as well as alerts by national authorities (Supplementary Figs. S2, S3 and S4). The routine use of the GPHF Minilab in faith-based DSOs has therefore successfully contributed to the WHO Global Surveillance and Monitoring System for SF Medical Products^2^.

Any medicines identified as probably falsified within the stock of the faith-based DSOs were quarantined and not distributed to patients, and deficient medicines offered by private suppliers to the DSOs were excluded from future drug procurement. Thereby, the project helped to prevent the spread of SF medicines in the health facilities supplied by the DSOs, and it increased the awareness of the problem of SF medicines. This is also evidenced by the low rate (0.3%) of probably falsified medicines found within the own stock of the involved DSOs.

The effects of the project extended even beyond the 100 APIs included in the GPHF Minilab manual: in 2020, the participating DSO from Chad discovered vitamin A capsules which failed visual inspection due to suspicious labelling mistakes. Compendial analysis at MEDS was initiated, and the products were found to be strongly degraded. The stated manufacturer confirmed that manufacturing and expiration date had been altered, apparently by criminals, to extend the stated product shelf life. WHO was informed and issued an international Medical Product Alert^50^. Furthermore, suspicious batches of carbamazepine tablets were reported by project partners in Cameroon. No Minilab monograph is available for this API, however the product failed a color reaction carried out according to the WHO handbook on basic test for pharmaceutical substances^51^. Subsequent compendial analysis conducted at MEDS showed absence of the declared API. The Cameroonian Ministry of Public Health was informed an issued an alert (Supplementary Fig. S4).

The medicine quality screening approach described here represents a very cost-effective way to contribute to the global surveillance for SF medical products. The total external funding of 49,600 € (Table 3) is extremely low compared to other international programs in health and/or in development cooperation. At the same time, this approach empowers local stakeholders to assume an active role in the surveillance for SF medicines. Of course, its limited sensitivity in the detection of substandard medicines must be kept in mind, and the GPHF Minilab can neither replace compendial analysis nor should it be used as a sole quality assurance (QA) measure in DSOs but rather as one part of a comprehensive QA system.

The Difäm-EPN Minilab Network operates since 2010 and has been growing constantly in this time. The success of this program depended especially on four factors: first, the existence of a well-established network of organizations with similar values and high commitment to the project. Second, the assurance of the continuous supply of the required consumables for GPHF Minilab analysis, funded by an external donor organization. Third, the possibility for confirmatory compendial analysis of suspected poor-quality samples, provided primarily by the WHO-prequalified medicine quality laboratory of MEDS, Nairobi, at moderate costs. The Certificates of Analysis provided by such a laboratory allow the involved DSOs to report their finding to national and international authorities, and to suppliers and manufacturers, as described in the present report. And fourth, the availability of an academically trained network coordinator (0.2 full-time equivalents) located at Difäm who supported organizational aspects, communications and training activities.

The role and the limitations of the GPHF Minilab in medicine quality assurance in low-resource settings need to be considered responsibly. The compliance with pharmacopeial standards can only be proven conclusively by (expensive) pharmacopeial methods. As mentioned, the GPHF Minilab is one of several available medicine quality screening techniques, and reviews of their respective strengths and limitations have been published^16–20,22^.

GPHF Minilab analysis is fast in comparison to full compendial analysis, but it still requires considerable staff time from the involved DSOs. It will certainly be worthwhile to further investigate the possibilities of a complementation of the use of the GPHF Minilab with the use of low-cost near-infrared and/or Raman spectroscopic devices^52^. Such devices hold promise for simple, inexpensive medicine quality screening and do not require consumables. However, spectral reference libraries still need to be created and maintained, and the possibilities and limitations of the application of spectroscopic devices in the quality screening of many different medicines from many different sources need to be investigated in field studies.

The involvement of private or civil society organizations, as described here, clearly offers the prospect to increase the outreach and speed of the detection and removal of SF medicines, especially in low-resource settings where government institutions may not be sufficiently equipped and staffed for the comprehensive completion of these tasks. However, such activities need to be carefully and diplomatically established, as government institutions may perceive them as an intrusion into their own responsibilities from the side of the civil society organizations. In fact, during the reporting period of the present study, one of the involved DSOs was informed by respective national Ministry of Health that the DSO is not authorized to control the quality of medicines. The appearance of a WHO Medical Product Alert about a falsified medicine in the respective country, based on a report from that DSO and a compendial analysis by MEDS, may have caused irritation in the government authorities. This DSO has since then stopped to share the results of their GPHF Minilab analyses with other stakeholders, which is a regrettable development for the Network. Possibly, institutions like the WHO can help to mediate a constructive dialogue between national authorities and civil society organizations to foster the development of a mutually beneficial and acceptable mode of cooperation in the surveillance for SF medicines.

For this study, data from 2019 and 2020 were evaluated, hence from the year before the outbreak of the COVID-19 pandemic, and from the first year of the pandemic. Notably, in 2020 ten falsified chloroquine products were found. This is most likely due to the “hype” of chloroquine and hydroxychloroquine as possible treatments for COVID-19, which strongly increased the demand and prices of these products^30,53–55^. Apparently, criminal falsifiers responded swiftly to this opportunity.

## Supplementary Information

Supplementary Information.

## Data Availability

The dataset generated during and analyzed during the current study are not publicly available. An anonymized version of the dataset is available from the corresponding author on reasonable request.

## References

[1] World Health Organization. *A Study on the Public Health and Socioeconomic Impact of Substandard and Falsified Medical Products*. (2017). https://www.who.int/publications/i/item/9789241513432.

[2] World Health Organization. *WHO Global Surveillance and Monitoring System for Substandard and Falsified Medical Products*. (2017). https://apps.who.int/iris/handle/10665/326708.

[3] Rahman MS (2018). The health consequences of falsified medicines: A study of the published literature. Trop. Med. Int. Health.

[4] Harris, J., Stevens, P. & Morris, J. Keeping it real. Combating the spread of fake drugs in poor countries. (2009). <https://www.africanliberty.org/wp-content/uploads/Keepingitreal.pdf>.

[5] Newton PN (2008). A collaborative epidemiological investigation into the criminal fake artesunate trade in South East Asia. PLoS Med..

[6] Newton PN, Caillet C, Guerin PJ (2016). A link between poor quality antimalarials and malaria drug resistance?. Expert Rev. Anti-Infect. Ther..

[7] Ayati N, Saiyarsarai P, Nikfar S (2020). Short and long term impacts of COVID-19 on the pharmaceutical sector. Daru.

[8] Tirivangani T, Alpo B, Kibuule D, Gaeseb J, Adenuga BA (2021). Impact of COVID-19 pandemic on pharmaceutical systems and supply chain: A phenomenological study. Explor. Res. Clin. Soc. Pharm..

[9] Aljadeed R (2021). The impact of COVID-19 on essential medicines and personal protective equipment availability and prices in Saudi Arabia. Healthcare.

[10] Bhaskar S (2020). At the epicenter of COVID-19: The tragic failure of the global supply chain for medical supplies. Front. Public Health.

[11] Bin Naeem S, Bhatti R, Khan A (2021). An exploration of how fake news is taking over social media and putting public health at risk. Health Inf. Libr. J..

[12] Newton PN (2020). COVID-19 and risks to the supply and quality of tests, drugs, and vaccines. Lancet Glob. Health.

[13] Interpol. *Global Operation Sees a Rise in Fake Medical Products Related to COVID-19*. (2020). https://www.interpol.int/News-and-Events/News/2020/Global-operation-sees-a-rise-in-fake-medical-products-related-to-COVID-19.

[14] World Health Organization. *Assessment of Medicines Regulatory Systems in Sub-Saharan African Countries. An Overview of Findings from 26 Assessment Reports*. (2010). http://apps.who.int/medicinedocs/en/d/Js17577en/.

[15] Hamilton WL, Doyle C, Halliwell-Ewen M, Lambert G (2016). Public health interventions to protect against falsified medicines: A systematic review of international, national and local policies. Health Policy Plan..

[16] Fadlallah R, El-Jardali F, Annan F, Azzam H, Akl EA (2016). Strategies and systems-level interventions to combat or prevent drug counterfeiting: A systematic review of evidence beyond effectiveness. Pharm. Med..

[17] Roth L, Biggs KB, Bempong DK (2019). Substandard and falsified medicine screening technologies. AAPS Open.

[18] Vickers S (2018). Field detection devices for screening the quality of medicines: A systematic review. BMJ Glob. Health.

[19] Zambrzycki SC (2021). Laboratory evaluation of twelve portable devices for medicine quality screening. PLoS Negl. Trop. Dis..

[20] Roth L, Nalim A, Turesson B, Krech L (2018). Global landscape assessment of screening technologies for medicine quality assurance: Stakeholder perceptions and practices from ten countries. Global. Health.

[21] Global Pharma Health Fund. *Global Use of the GPHF-Minilab™*. (2021). https://www.gphf.org/en/minilab/einsatzgebiete.htm.

[22] U.S. Pharmacopeial Convention. *USP Technology Review: Global Pharma Health Fund (GPHF): Minilab™*. https://www.usp.org/sites/default/files/usp/document/our-work/global-public-health/2020-usp-technology-review-global-pharma-health-fund-minilab.pdf (2020).

[23] Jähnke, R. W. O. & Dwornik, K. *A Concise Quality Control Guide On Essential Drugs And Other Medicines: Review And Extension*. 3 edn, (Global Pharma Health Fund, 2020).

[24] Schäfermann S (2020). Substandard and falsified antibiotics and medicines against noncommunicable diseases in western Cameroon and northeastern Democratic Republic of Congo. Am. J. Trop. Med. Hyg..

[25] Asia Development Bank, I. D. D. O., Mahidol-Oxford Research Unit & Georgia Tech. *An Evaluation of Portable Screening Devices to Assess Medicines Quality for National Medicines Regulatory Authorities*. (2018). https://www.iddo.org/external-publication/evaluation-portable-screening-devices-assess-medicines-quality-national.

[26] Petersen A, Held N, Heide L, Difam-EPN-Minilab Survey Group (2017). Surveillance for falsified and substandard medicines in Africa and Asia by local organizations using the low-cost GPHF Minilab. PLoS ONE.

[27] Global Pharma Health Fund. *GPHF-Minilab™: Main Manual Now Updated and Extended.* https://www.gphf.org/en/minilab/manuals.htm (2022).

[28] WHO Expert Committee on Specifications for Pharmaceutical Preparations. *WHO Expert Committee On Specifications For Pharmaceutical Preparations. Forty-eigth Report.* Ch. Annex 3. A model quality assurance system for procurement agencies, 137–291 (World Health Organization, 2014).

[29] Degardin K, Roggo Y, Margot P (2014). Understanding and fighting the medicine counterfeit market. J. Pharm. Biomed. Anal..

[30] Gnegel G (2020). Identification of falsified chloroquine tablets in Africa at the time of the COVID-19 pandemic. Am. J. Trop. Med. Hyg..

[31] Hauk C, Hagen N, Heide L (2021). Identification of substandard and falsified medicines: influence of different tolerance limits and use of authenticity inquiries. Am. J. Trop. Med. Hyg..

[32] African Union. *Member States*. (2022). https://au.int/en/member_states/countryprofiles2.

[33] MedCalc Software Ltd. *Comparison of Proportions Calculator, Version 20.014*. https://www.medcalc.org/calc/comparison_of_proportions.php. Accessed 28 Oct 2021.

[34] Richardson JT (2011). The analysis of 2 x 2 contingency tables: Yet again. Stat. Med..

[35] Campbell I (2007). Chi-squared and Fisher-Irwin tests of two-by-two tables with small sample recommendations. Stat. Med..

[36] World Health Organization. *Medical Product Alert N° 6/2019: Falsified Hydrochlorothiazide (Containing Glibenclamide) in Cameroon*. (2019). https://www.who.int/news/item/17-04-2019-medical-product-alert-n-6-2019-(english-version).

[37] World Health Organization. *Medical Product Alert N°10/2019: Falsified Quinine Bisulphate Circulating in Uganda and Quinine Sulphate Circulating in Central African Republic and Chad*. (2019). https://www.who.int/news/item/16-10-2019-medical-product-alert-n-10-2019-(english-version.

[38] Khuluza F, Kigera S, Heide L (2017). Low prevalence of substandard and falsified antimalarial and antibiotic medicines in public and faith-based health facilities of southern Malawi. Am. J. Trop. Med. Hyg..

[39] Ali GKM, Ravinetto R, Alfadl AA (2020). The importance of visual inspection in national quality assurance systems for medicines. J. Pharm. Policy Pract..

[40] World Health Organization. *Medical Product Alert N°4/2020: Falsified Chloroquine (Update)*. (2020). https://www.who.int/news/item/09-04-2020-medical-product-alert-n4-2020.

[41] World Health Organization. *Medical Product Alert N° 1/2020: Falsified antimalarials displaying an outdated WHO Essential Drugs Programme logo*. (2020). https://www.who.int/news/item/09-03-2020-medical-product-alert-n-1-2020-english-version.

[42] NAFDAC. *Public Alert no. 0009/2019: Alert on Falsified Hydrochlorothiazide 50mg (Containing Glibenclamide) Circulating in Cameroon*. (2019). https://www.nafdac.gov.ng/public-alert-no-0009-2019-alert-on-falsified-hydrochlorothiazide-50mg-containing-glibenclamide-circulating-in-cameroon/.

[43] NAFDAC. *Public Alert No. 004/2020: Alert on Falsified Chloroquine Phosphate 250 mg Tablets Circulating in Cameroon*. (2020). https://www.nafdac.gov.ng/public-alert-no-004-2020-alert-on-falsified-chloroquine-phosphate-250mg-tablets-circulating-in-cameroon/.

[44] NAFDAC. *Public Alert No. 005/2020: Alert on Falsified Chloroquine Products Circulating in WHO Region of Africa*. (2020). https://www.nafdac.gov.ng/public-alert-no-005-2020-alert-on-falsified-chloroquine-products-circulating-in-who-region-of-africa/.

[45] Drug Regulatory Authority of Pakistan. *Alert of Falsified Quinine Bisulphate: Circulating in Uganda and Quinine Sulphate Circulating in Central African Republic and Chad*. (2019). https://www.dra.gov.pk/docs/SAFETY%20ALERT%20OF%20FALSIFIED%20QUININE.pdf.

[46] NAFDAC. *Public Alert No. 012/2020: Presence of Suspected Falsified SA’A TRIM (Sulfamethoxazole) Circulating in an Illicit Market in Chad*. (2020). https://www.nafdac.gov.ng/public-alert-no-012-2020-presence-of-suspected-falsified-saa-trim-sulfamethoxazole-circulating-in-an-illicit-market-in-chad/.

[47] Rahman MS (2022). A comprehensive analysis of selected medicines collected from private drug outlets of Dhaka city, Bangladesh in a simple random survey. Sci. Rep..

[48] Seitzer M, Klapper S, Mazigo HD, Holzgrabe U, Mueller A (2021). Quality and composition of albendazole, mebendazole and praziquantel available in Burkina Faso, Cote d'Ivoire, Ghana and Tanzania. PLoS Negl. Trop. Dis..

[49] Ozawa S (2018). Prevalence and estimated economic burden of substandard and falsified medicines in low- and middle-income countries: A systematic review and meta-analysis. JAMA Netw. Open.

[50] World Health Organization. *Medical Product Alert N°1/2021: Falsified vitamin A*. (2021). https://www.who.int/news/item/10-03-2021-medical-product-alert-n-1-2021-falsified-vitamin-a.

[51] World Health Organization. *Basic Tests for Pharmaceutical Substances*. (1986). https://apps.who.int/iris/handle/10665/39594.

[52] Luangasanatip N (2021). Implementation of field detection devices for antimalarial quality screening in Lao PDR: A cost-effectiveness analysis. PLoS Negl. Trop. Dis..

[53] Sulis G, Batomen B, Kotwani A, Pai M, Gandra S (2021). Sales of antibiotics and hydroxychloroquine in India during the COVID-19 epidemic: An interrupted time series analysis. PLoS Med..

[54] Haque M (2021). Changes in availability, utilization, and prices of medicines and protection equipment for COVID-19 in an urban population of northern Nigeria. J. Res. Pharm. Pract..

[55] Sefah IA (2020). Rapid assessment of the potential paucity and price increases for suggested medicines and protection equipment for COVID-19 across developing countries with a particular focus on Africa and the implications. Front. Pharmacol..

