# Supplementary material for: Surveillance for substandard and falsified medicines by local faith-based organizations in 13 low- and middle-income countries using the GPHF Minilab
Source: Sci Rep. 2022 Jul 30;12:13095. doi: 10.1038/s41598-022-17123-0 (PMC9338985; doi:10.1038/s41598-022-17123-0)
Supplement: Supplementary file 1 — Supplementary Information. [file 41598_2022_17123_MOESM1_ESM.pdf]

Surveillance for substandard and falsified medicines by local faith-based organizations in 13 low- and middle-income countries using the GPHF Minilab

Supplementary Information

Gesa Gnegel[1,2], Christine Häfele-Abah[2,3], Richard Neci[3], Difäm-EPN Minilab Network[2,3], and Lutz Heide[1,3]*

[1] Pharmaceutical Institute, Eberhard Karls University Tuebingen, Tuebingen, Germany

[2] German Institute for Medical Mission (Difäm), Tübingen, Germany

[3] Ecumenical Pharmaceutical Network (EPN), Nairobi, Kenya

*heide@uni-tuebingen.de

[redacted] & LABS. NIG. LTD

RE: PRODUCT RECALL/ PRODUCT SAFETY INFORMATION CONCERNS PRODUCT: ZIMATRIM TABLET

Dear [redacted]

We would like to notify you about urgent correction measure with the users of Zimatrim tablets which was confirmed to have intra batch deviation in hardness of the tablets affecting the disintegration of the tablet.

This has been initiated for the batch Number listed below

ZCT 027

ZCT 022

According to our files, two of the listed batches of Zimatrim was delivered to you and it is therefore involved in this action

[redacted] [redacted]

Yours Sincerely,

[redacted]

Head of Quality Management.

Supplementary Figure S1: Product recall issued by a Nigerian manufacturer

MINISTERE DE LA SANTE ET DE LA POPULATION
****
DIRECTION DE CABINET
****
INSPECTION CENTRALE DES SERVCES PHARMACEUTIQUES ET LABORATOIRES
****
N° [illegible] /MSP/DIRCAB/ICSPL.

REPUBLIQUE CENTRAFRICAINE
Unité – Dignité – Travail
****

Date : 27 SEPT 2019

COMMUNIQUE DE PRESSE DU

MINISTRE DE LA SANTE ET DE LA POPULATION

Objet : CIRCULATION DE QUININE SULFATE FALSIFIE comprimé

Le Ministre de la Santé et de la Population sollicite la plus grande vigilance des professionnels de la santé et du grand public concernant la circulation d'une version falsifié de lots de QUININE SULFATE comprimé.

Ces produits ont été découverts dans les centres de santé des districts sanitaire du Bangassou et Bossangoa.

Les détails sur le produits sont les suivants ;

N°	1	2	3
Nom du produit	QUININE SULPHATE 800 mg	QUININE SULPHATE 300 mg	QUININE SULPHATE 300 mg
Fabricant	Pharmachim bulgaria	Pharmachim bulgaria	Laboratory & Allied ltd
numéro de lot	00952005	7711006	7422
Date de fabrication	06/2015	08/2018	03-2017
Date de péremption	12/2020	7/2021	04-2021

Le Ministre de la Santé et de la Population recommande aux médecins chefs des districts précités de procéder , dès la réception de ce communiqué, immédiatement au retrait et la mise en quarantaine desdits produits dans les centres de santé de leurs juridictions.

Le Ministre de la Santé et de la Population

MINISTERE DE LA SANTE ET DE LA POPULATION – Le Ministre

Dr Pierre SOMSE

MINISTERE DE LA SANTE ET DE LA POPULATION
****
DIRECTION DE CABINET
****
INSPECTION CENTRALE DES SERVCES PHARMACEUTIQUES ET LABORATOIRES
****
N° [illegible] /MSP/DIRCAB/ICSPL.

REPUBLIQUE CENTRAFRICAINE
Unité – Dignité – Travail
****

Date : 27 SEPT 2019

INSTRUCTION MINISTERIELLE

Suite à la notification de l'alerte internationale de l'OMS réf : **N° 10/2019 relative à des lots de QUININE SULPHATE FALSIFIES** circulant sur le territoire centrafricain, le Ministre de la Santé et de la Population instruit tous les médecins chefs de district de procéder à une recherche active de ces différents lots dans toutes les formations sanitaires de leurs juridictions afin de procéder à leur retrait et mise en quarantaine et d'en informer la direction de la pharmacie et du médicament.

-Copie DPLMT pour suivi

Le Ministre de la Sante et de la Population

MINISTERE DE LA SANTE ET DE LA POPULATION – Le Ministre

Dr Pierre SOMSE

Supplementary Figure S2: National medical product alert published by the Ministry of Health and Population of the Central African Republic

REPUBLIQUE DU CAMEROUN
Paix-Travail-Patrie

Laboratoire National de Contrôle de Qualité des Médicaments et d'Expertise
----
DIRECTION GENERALE

REPUBLIC OF CAMEROON
Peace-Work-Fatherland

National Drug Quality Control and Valuation Laboratory

GENERAL MANAGEMENT

LANACOME
Laboratoire National de Contrôle de Qualité des Médicaments et d'Expertise
When Passion for Quality leads to Integrity

Réf : 1310 /20/L/LANACOME/DG/.- *Yaoundé, le* 30 MARS 2020

LE DIRECTEUR GENERAL

1) Aux Directeurs des:
- ***Hôpitaux Généraux,***
- ***Hôpitaux Centraux,***
- ***Hôpitaux de District***
- ***Cliniques privées***

2) Aux Pharmaciens d'Officines et Pharmaciens Chefs des Hôpitaux et Cliniques privées
3) Aux Grossistes agréés

Objet : Circulation de la Chloroquine sans principe actif.

Mesdames et Messieurs les Directeurs des établissements hospitaliers,

Le Laboratoire National de Contrôle de Qualité des Médicaments et d'Expertise informe les populations et les professionnels de la santé que deux présentations de chloroquine issue des circuits de contrebande sont actuellement en circulation au Cameroun et se retrouveraient déjà dans certaines formations sanitaires.

Les résultats des tests de ces deux présentations de chloroquine ci-dessous révèlent l'absence de toute substance active pharmaceutique.

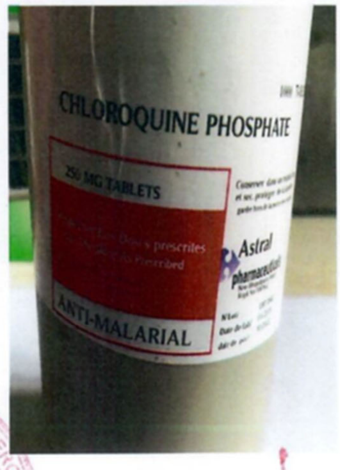

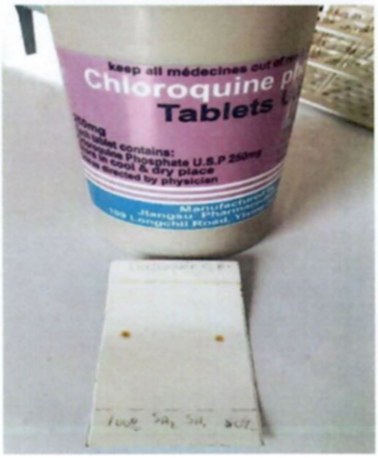

LANACOME, Rue Rudolph Manga Bell, B.P. 12 216 Yaoundé ; Tél. 237 22 60 43 02, Fax. 237 22 23 93 50/ 237 22 23 06 60, email :contact@lanacome.cm / site web : www.lanacome.cm 1
Etablissement public administratif crée par décret N° 96/055 du 12 mars 1996 et Réorganisé comme établissement publique à caractère scientifique et technique par décret N°764/2018 du 11 décembre 2018.

Supplementary Figure S3: National medical product alert published by the National Drug Quality Control and Valuation Laboratory of Cameroun

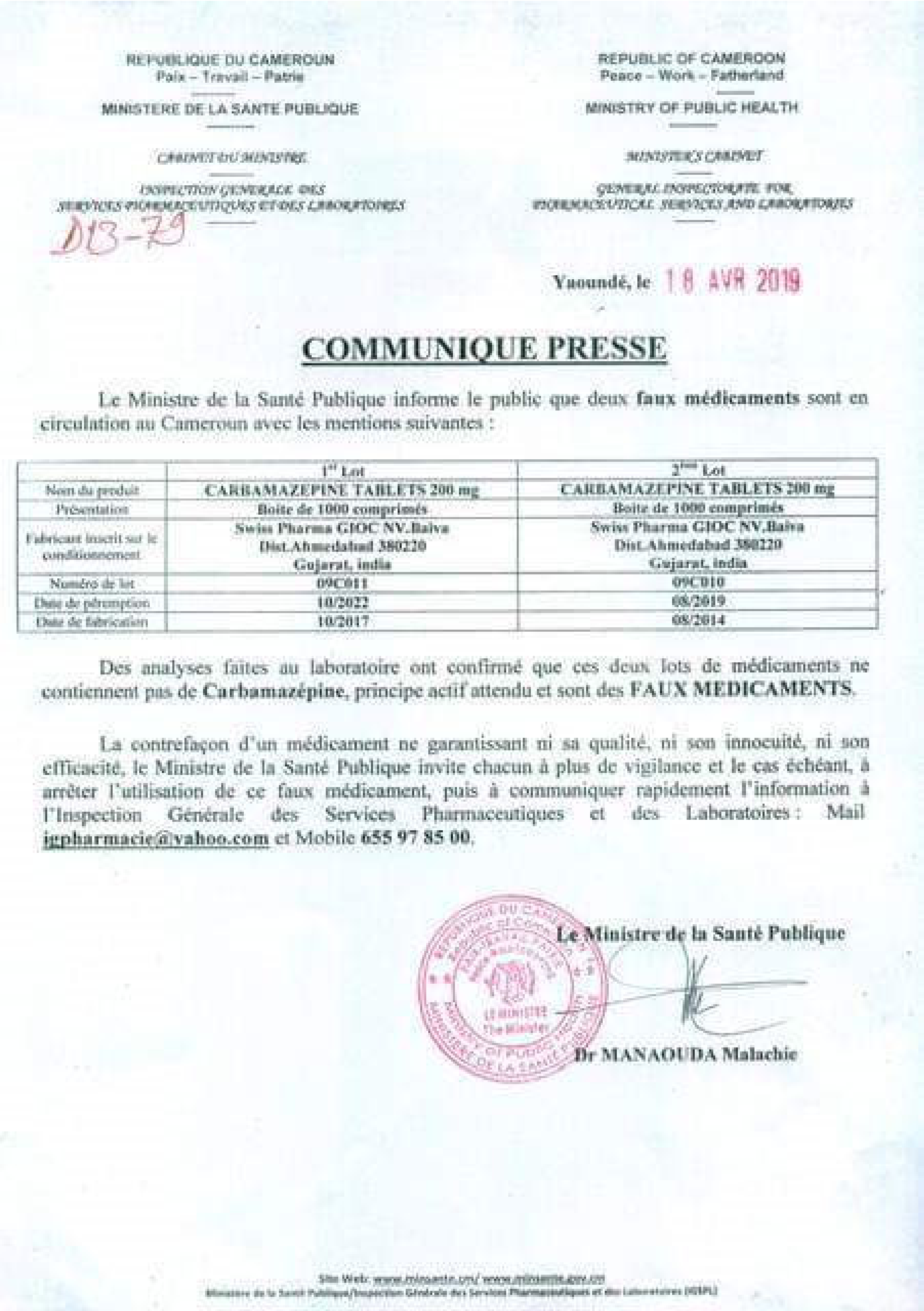

REPUBLIQUE DU CAMEROUN
Paix – Travail – Patrie

MINISTERE DE LA SANTE PUBLIQUE

CABINET DU MINISTRE

INSPECTION GENERALE DES SERVICES PHARMACEUTIQUES ET DES LABORATOIRES

D13-79

REPUBLIC OF CAMEROON
Peace – Work – Fatherland

MINISTRY OF PUBLIC HEALTH

MINISTER'S CABINET

GENERAL INSPECTORATE FOR PHARMACEUTICAL SERVICES AND LABORATORIES

Yaoundé, le 18 AVR 2019

COMMUNIQUE PRESSE

Le Ministre de la Santé Publique informe le public que deux **faux médicaments** sont en circulation au Cameroun avec les mentions suivantes :

	1er Lot	2ème Lot
Nom du produit	**CARBAMAZEPINE TABLETS 200 mg**	**CARBAMAZEPINE TABLETS 200 mg**
Présentation	**Boite de 1000 comprimés**	**Boite de 1000 comprimés**
Fabricant inscrit sur le conditionnement	**Swiss Pharma GIOC NV.Baiva Dist.Ahmedabad 380220 Gujarat, india**	**Swiss Pharma GIOC NV.Baiva Dist.Ahmedabad 380220 Gujarat, india**
Numéro de lot	**09C011**	**09C010**
Date de péremption	**10/2022**	**08/2019**
Date de fabrication	**10/2017**	**08/2014**

Des analyses faites au laboratoire ont confirmé que ces deux lots de médicaments ne contiennent pas de **Carbamazépine**, principe actif attendu et sont des **FAUX MEDICAMENTS**.

La contrefaçon d'un médicament ne garantissant ni sa qualité, ni son innocuité, ni son efficacité, le Ministre de la Santé Publique invite chacun à plus de vigilance et le cas échéant, à arrêter l'utilisation de ce faux médicament, puis à communiquer rapidement l'information à l'Inspection Générale des Services Pharmaceutiques et des Laboratoires : Mail **igpharmacie@yahoo.com** et Mobile **655 97 85 00**.

Le Ministre de la Santé Publique

Dr MANAOUDA Malachie

[illegible]

Supplementary Figure S4: National medical product alert published by the Cameroonian Ministry of Health

API	Total number of samples	Number of stated manufacturers	Stated strength	Dosage Form	Number of samples	Probably substandard samples	Probably falsified samples
Acetylsalicylic acid	22	13	75mg	tablet	4		
			81mg	tablet	5		
			100mg	tablet	2		
			300mg	tablet	3		
			500mg	tablet	6	3	
			500mg	injection	1		
			1000mg	injection	1		
Acetylsalicylic acid + paracetamol	6	2	400mg	tablet	3		
			325+400mg	tablet	1		
			200+400mg	tablet	1		
			300+600mg	tablet	1		
Aciclovir	9	4	400mg	tablet	6		
			800mg	tablet	3		
Albendazole	36	26	200mg	tablet	1		
			400mg	tablet	35	1	
Aminophylline	19	11	100mg	tablet	15		
			250mg/10mL	injection	4		
Amlodipine	50	30	5mg	capsule	1		
			5mg	tablet	23		
			10mg	tablet	26		
Amodiaquine	1	1	200mg	tablet	1		
Amoxicilline	101	49	125mg	tablet	1		
			250mg	tablet	17	3	
			500mg	tablet	15	1	
			250mg	capsule	24	1	
			500mg	capsule	36	1	
			1000mg	injection	1		
			125mg/5ml	dry syrup	2		
			250mg/5ml	dry syrup	5		
Amoxicilline + clavulanic acid	47	33	250+125mg	capsule	1		
			500+125mg	capsule	2		
			1000+200mg	injection	4		
			250+125mg	tablet	2		
			500+62.5mg	tablet	1		
			500+125mg	tablet	25		
			875+125mg	tablet	5	1	
			200+28.5mg/5ml	dry syrup	2		
			250+31.25mg/5ml	dry syrup	1		
			250+62.5mg/5ml	dry syrup	4		
Ampicilline	15	8	500mg	injection	1		
			1000mg	injection	14		
Ampicilline + cloxacilline	6	5	250+250mg	capsule	5		1
			250+250mg	tablet	1		
Artemether	26	20	20mg	injection	4		
			40mg	injection	2		
			80mg	injection	20		
Artemether + lumefantrine	130	62	20+120mg	tablet	66		1
			40+240mg	tablet	1		
			80+480mg	tablet	43		
			15+90mg/5ml	dry syrup	10		
			20+120mg/5ml	dry syrup	10		
Artesunate	28	15	60mg	injection	27		
			120mg	injection	1		

API	Total number of samples	Number of stated manufacturers	Stated strength	Dosage Form	Number of samples	Probably substandard samples	Probably falsified samples
Artesunate + amodiaquine	2	2	100+270mg	tablet	2		
Atenolol	25	16	25mg	tablet	3		
			50mg	tablet	16		
			100mg	tablet	6		
Azithromycin	51	31	250mg	capsule	2		
			250mg	tablet	14		
			500mg	tablet	35		
Benzylpenicillin benzathine	16	6	1000mg	injection	3		
			2.4 mega	injection	13		
Benzylpenicillin procain	1	1	3 mio IU	injection	1		
Benzylpenicillin sodium	2	2	1 mega	injection	2		
Bisoprolol	1	1	10mg	tablet	1		
Captopril	14	9	25mg	tablet	14	4	
Cefalexin	9	7	250mg	capsule	4		
			500mg	capsule	5	1	
Cefixime	42	28	100mg	tablet	2		
			200mg	tablet	23		
			400mg	tablet	16		
			500mg	tablet	1		
Cefotaxime	4	1	1000mg	injection	4		
Cefpodoxime	1	1	200mg	tablet	1		
Ceftriaxone	66	32	250mg	injection	1		
			500mg	injection	1		
			1000mg	injection	64		3
Cefuroxime	3	2	250mg	tablet	1		
			500mg	tablet	2		
Cetirizine	5	4	10mg	tablet	5		
Chloramphenicol	10	7	250mg	capsule	10		
Chloroquine	45	15	100mg	tablet	11	1	5
			250mg	tablet	34		5
Chlorphenamine	16	12	4mg	tablet	16		
Ciprofloxacin	85	52	200mg/100ml	injection	3		
			250mg	tablet	3		
			500mg	tablet	77	2	
			750mg	tablet	1		
			500mg	capsule	1		
Clarithromycin	14	10	500mg	tablet	14		
Clindamycin	3	3	300mg	capsule	2		
			300mg	tablet	1		
Clomifene	7	4	50mg	tablet	7		
Cloxacillin	45	18	1000mg	injection	1	1	
			500mg	injection	7		
			250mg	capsule	13		
			500mg	capsule	21	1	
			500mg	tablet	3		
Dapsone	3	1	100mg	tablet	3		
Diclofenac	51	40	25mg	injection	1		
			75mg	injection	9		
			50mg	tablet	30	4	
			100mg	tablet	11	1	
Diclofenac + paracetamol	5	5	50+325mg	tablet	1		
			50+500mg	tablet	4		1
Diclofenac + paracetamol + chlorphenamine	1	1	50+500+4mg	tablet	1		

API	Total number of samples	Number of stated manufacturers	Stated strength	Dosage Form	Number of samples	Probably substandard samples	Probably falsified samples
Dihydroartemisinin + piperaquine	7	4	40+320mg	tablet	7		
Doxycycline	37	13	100mg	capsule	11	1	
			100mg	tablet	25		
			200mg	tablet	1		
Erythromycin	34	21	250mg	tablet	17	3	
			500mg	tablet	17		
Fluconazole	10	9	200mg	capsule	6		
			150mg	tablet	1		
			200mg	tablet	2		
			200mg/100ml	injection	1		
Furosemide	34	23	20mg/ml	injection	1		
			20mg/2ml	injection	4		
			40mg	tablet	29		
Gentamicin	12	11	80mg/2ml	injection	12		
Glibenclamide	27	16	5mg	tablet	27		
Griseofulvin	16	9	250mg	tablet	6		
			500mg	tablet	10		
Hydrochlorothiazide	26	13	25mg	tablet	18		
			50mg	tablet	8	1	1
Levofloxacin	32	25	250mg	tablet	1		
			500mg	tablet	30		
			750mg	tablet	1		
Lisinopril	12	5	5mg	tablet	1		
			10mg	tablet	1		
			20mg	tablet	10		
Mebendazole	27	16	100mg	tablet	23	1	
			400mg	tablet	1		
			500mg	tablet	2		
			500mg/100ml	injection	1		
Mefenamic acid	2	1	250mg	tablet	1		
			500mg	tablet	1		
Metformin	59	35	500mg	tablet	45	2	
			750mg	tablet	1		
			850mg	tablet	9		
			1000mg	tablet	4		
Metoclopramide	8	8	10mg	tablet	7		
			10mg	injection	1		
Metronidazole	66	41	500mg/100ml	injection	4		
			200mg	tablet	22		
			250mg	tablet	28	1	
			400mg	tablet	5		
			500mg	tablet	7	1	
Naproxen	2	2	500mg	tablet	2		
Nifedipine	26	20	10mg	tablet	4		
			20mg	tablet	20		
			25mg	tablet	1		
			30mg	tablet	1		
Ofloxacin	13	10	200mg	injection	1		
			200mg	tablet	8		
			400mg	tablet	4		
Omeprazole	34	25	20mg	capsule	22		
			20mg	tablet	1		
			40mg	injection	11		

API	Total number of samples	Number of stated manufacturers	Stated strength	Dosage Form	Number of samples	Probably substandard samples	Probably falsified samples
Paracetamol	147	64	400mg	capsule	1		
			10mg/ml	injection	2		
			300mg/2ml	injection	2		
			1000mg/100ml	injection	8		
			100mg	tablet	8		
			300mg	tablet	1		
			500mg	tablet	125	6	
Phenoxymethylpenicillin	23	6	250mg	tablet	21	1	
			500mg	tablet	2		
Piperaquine	1	1	375mg	tablet	1	1	
Praziquantel	4	3	600mg	tablet	4		
Prednisolone	50	26	5mg	tablet	50	2	
Proguanil	5	4	100mg	tablet	5		1
Quinine	74	39	300mg/ml	injection	1		
			500mg	injection	3		
			600mg/2ml	injection	7		
			100mg	tablet	9		
			300mg	tablet	41	2	7
			400mg	tablet	1		
			500mg	tablet	11	1	
			800mg	tablet	1		1
Ranitidine	6	4	50mg	injection	1		
			150mg	tablet	1		
			300mg	tablet	4		
Salbutamol	7	6	2mg	tablet	2		
			4mg	tablet	5		
Simvastatin	5	3	40mg	tablet	5		
Sulfadoxine + pyrimethamine	20	13	500+25mg	tablet	20	1	
Sulfamethoxazole + trimethoprim	68	36	100+20mg	tablet	6		
			400+80mg	tablet	55	4	8
			800+160mg	tablet	7		
Tetracycline	2	2	250mg	tablet	1		
			250mg	capsule	1		
**Total**	**1919**	**495**			**1919**	**54**	**34**

Supplementary Table S1: Overview of all samples included in this study

No.	Country of discovery	Declared active pharmaceutical ingredient (API)	Name of product	Batch no	Expiry date	Stated manufacturer	Stated country of origin	Quality deficiency
1	Cameroon	Ampicillin trihydrate, Cloxacillin sodium	Amcloxin 250/250mg cps	A015050	04.20	MAXHEAL Pharmaceuticals(India)Ltd.	India	declared API absent
2	Chad	Artemether, Lumefantrine	COMBIART 20/120mg tbl	7225119 (carton) 7225500 (blister)	08/2021	Strides ARCOLAB LIMITED	India	declared API absent
3	DR Congo	Ceftriaxone sodium	Ceftriaxone 1g inj	J-104	06/2021	LDP – Laboratoirios TORLAN S.A	Spain	API content 23.5 % of stated content
4	DR Congo	Ceftriaxone sodium	Ceftriaxone 1g inj	J-104	06/2020	LDP - Laboratoirios TORLAN S.A	Spain	API content 23.8 % of stated content
5	DR Congo	Ceftriaxone sodium	Ceftriaxone 1g inj	M-63	01/2022	LDP - Laboratoirios TORLAN S.A	Spain	total vial content less than 230 mg
6	DR Congo	Chloroquine	CLOROQUINE 250mg tbl	1605059	04/ 2023	Dawa Limited	Kenya	declared API absent; 126.5 mg metronidazole
7	Cameroon	Chloroquine phosphate	CHLOROQUINE PHOSPHATE 250mg tbl	EBT 2512	10/2022	Astral pharmaceuticals	India	declared API absent
8	Nigeria	Chloroquine phosphate	ENI-QUIN 250mg tbl	CQLL	July 2023	ENITOP PHARMACEUTICAL Nig. L	Nigeria	declared API absent
9	Cameroon	Chloroquine phosphate	CHLOROQUINE PHOSPHATE 100mg tbl	h-659	07/04/21	Enitop Pharmaceutical Nig. Ltd.	Nigeria	declared API absent
10	Cameroon	Chloroquine phosphate	Chloroquine Phosphate 100mg tbl	660	08/2022	Jiangsu Pharmaceuticals Inc.	China	API content 21.7 % of stated content
11	Cameroon	Chloroquine phosphate	Chloroquin Phosphate 100mg tbl	660	04/2023	Jiangsu Pharmaceuticals Inc.	China	declared API absent; 14.1 mg metronidazole
12 &13	Cameroon	Chloroquine phosphate	Chloroquine Phosphate 100mg tbl	660	05/2021	Jiangsu Pharmaceuticals Inc.	China	declared API absent; 35.7 mg paracetamol
14	Cameroon	Chloroquine phosphate	Chloroquine phosphate 250mg tbl	660	09/2022	Jlangsu Pharmaceuticals Inc.	China	declared API absent; 14.6mg metronidazole 1.6 mg paracetamol
15	Cameroon	Chloroquine phosphate	SA'A QUINE 250mg tbl	SQ 19024	10/2022	SA'A Pharmaceutical Products Limited.	Nigeria	API content 12.2 % of stated content

No.	Country of discovery	Declared active pharmaceutical ingredient (API)	Name of product	Batch no	Expiry date	Stated manufacturer	Stated country of origin	Quality deficiency
16	Cameroon	Hydrochlorothiazide	HYDROCHLOROTHIAZIDE 50mg tbl	16G04	30/05/2021	LABORATOIRES STEROP	Belgium	declared API absent; 5mg glibenclamide
17	Cameroon	Paracetamol, Diclofenac sodium	Gabamol 500/50mg tbl	MP9820	09/2023	McCoy Pharma Pvt. Ltd.	India	declared API diclofenac absent (95.2% paracetamol)
18	Nigeria	Proguanil	PROGUANIL 100mg tbl	P.626	05 / 2021	PHARMAMED	Malta	only traces of API contained
19	DR Congo	Quinine	QUININE 300mg tbl	T459Q	10/2022	-	India	declared API absent
20	Chad	Quinine	QUININE SULPHATE 300mg tbl	7711006	7/2021	Enitop Pharmaceutical Nig. Ltd.	Nigeria	declared API absent
21	Central Afr. Rep.	Quinine sulphate	QUININE SULPHATE 300mg tbl	7711006	7/2021	Enitop Pharmaceutical Nig. Ltd.	Nigeria	declared API absent
22	Central Afr. Rep.	Quinine sulphate	QUININE SULPHATE 300mg tbl	7711006	05/2022	Enitop Pharmaceutical Nig. Ltd.	Nigeria	declared API absent
23	Central Afr. Rep.	Quinine sulphate	QUININE SULPHATE 800mg tbl	00952005	12/2020	Pharmachim	Bulgaria	declared API absent
24	Chad	Quinine sulphate	QUININE SULPHATE 300mg tbl	44680	04/ 2021	Remedica Ltd	Cyprus	declared API absent; 12 mg chloroquine
25	DR Congo	Quinine sulphate	Quinine Sulphate 300mg tbl	022157	10/2021	RENE INDUSTRIES LTD.	Uganda	declared API absent
26	Chad	Quinine sulphate	QUININE SULPHATE 300mg tbl	8858	-	WEIDER FARMASOYTSIKE A/S	Norway	declared API absent
27	Chad	Sulfamethoxazole + Trimethoprim	Arveltrim 80/400mg tbl	A.M 420	06/22	Arvel Marris Pharmaceutical Nig Ltd.	Nigeria	declared API absent
28	Nigeria	Sulfamethoxazole + Trimethoprim	GILTRIM 80/80mg tbl	SCT:002	Jan. 2023	GIL INDUSTRIES LTD.	Nigeria	declared API absent
29	Chad	Sulfamethoxazole + Trimethoprim	KOLLYTRIM 80/400mg tbl	-	-	KOLLINTON PHARMACEUTICAL IND. LTD.	-	declared APIs absent; small amount paracetamol
30	Chad	Sulfamethoxazole + Trimethoprim	MEDITRIM 80/400mg tbl	C 10M	03/23	MEDIVILLE PHARMACEUTICAL NIG. LTD.	Nigeria	declared API absent
31	Chad	Sulfamethoxazole + Trimethoprim	Optrim 80/400mg tbl	OLL20	12/23	OPTIMAL LAB LTD.	Nigeria	declared API absent

No.	Country of discovery	Declared active pharmaceutical ingredient (API)	Name of product	Batch no	Expiry date	Stated manufacturer	Stated country of origin	Quality deficiency
32	Chad	Sulfamethoxazole + Trimethoprim	POLETRIM 80/400mg tbl	409.38	07/23	MAOBISON INTER – Link & ASSOCIATES LTD.	Nigeria	sulfamethoxazole 47.7 % trimethoprim 21.2 % of stated content
33	Chad	Sulfamethoxazole + Trimethoprim	SA'A TRIM 80/400mg tbl	ST191097	10/2022	SA'A Pharmaceutical Products Ltd	Nigeria	sulfamethoxazole 17.6 % trimethoprim 16.3 % of stated content
34	Chad	Sulfamethoxazole + Trimethoprim	SA'A TRIM 80/400mg tbl	-	-	SA'A Pharmaceutical Products Ltd	Nigeria	API content less than 25% (see sample above)

Supplementary Table S2: Medicine samples identified in this study as probably falsified.
In the cases no. 12 & 13, two samples of this medicine were identified independently in the course of this study.
